# Ultrasound Molecular Imaging as a Potential Non-invasive Diagnosis to Detect the Margin of Hepatocarcinoma via CSF-1R Targeting

**DOI:** 10.3389/fbioe.2020.00783

**Published:** 2020-07-14

**Authors:** Qiongchao Jiang, Yunting Zeng, Yanni Xu, Xiaoyun Xiao, Hejun Liu, Boyang Zhou, Yao Kong, Phei Er Saw, Baoming Luo

**Affiliations:** ^1^Department of Ultrasound, Sun Yat-sen Memorial Hospital, Sun Yat-sen University, Guangzhou, China; ^2^Guangdong Provincial Key Laboratory of Malignant Tumor Epigenetics and Gene Regulation, Sun Yat-sen Memorial Hospital, Sun Yat-sen University, Guangzhou, China; ^3^Department of Hyperbaric Oxygen, Sun Yat-sen Memorial Hospital, Sun Yat-sen University, Guangzhou, China; ^4^Guangdong Provincial Key Laboratory of Malignant Tumor Epigenetics and Gene Regulation, Medical Research Center, Sun Yat-sen Memorial Hospital, Sun Yat-sen University, Guangzhou, China

**Keywords:** ultrasound imaging, HCC tumor margin, non-invasive tumor margin detection, CSF-1R targeting, macrophage

## Abstract

Though radiofrequency ablation (RFA) is considered to be an effective treatment for hepatocellular carcinoma (HCC), but more than 30% of patients may suffer insufficient RFA (IRFA), which can promote more aggressive of the residual tumor. One possible method to counter this is to accurately identify the margin of the HCC. Colony-stimulating factor 1 receptor (CSF-1R) has been found to be restrictively expressed by tumor associated macrophages (TAMs) and monocytes which more prefer to locate at the boundary of HCC. Using biotinylation method, we developed a CSF-1R-conjugated nanobubble CSF-1R (NB_CSF–1R_) using a thin-film hydration method for margin detection of HCC. CSF-1R expression was higher in macrophages than in HCC cell lines. Furthermore, immunofluorescence showed that CSF-1R were largely located in the margin of xenograft tumor and IFRA models. *In vitro*, NB_CSF–1R_ was stable and provided a clear ultrasound image even after being stored for 6 months. In co-culture, NB_CSF–1R_ adhered to macrophages significantly better than HCC cells (*p* = 0.05). In *in vivo* contrast-enhanced ultrasound imaging, the washout half-time of the NB_CSF–1R_ was significantly greater than that of NB_CTRL_ and Sonovue^®^ (*p* = 0.05). The signal intensity of the tumor periphery was higher than the tumor center or non-tumor region after NB_CSF–1R_ injection. Taken together, NB_CSF–1R_ may potentially be used as a non-invasive diagnostic modality in the margin detection of HCC, thereby improving the efficiency of RFA. This platform may also serve as a complement method to detect residual HCC after RFA; and may also be used for targeted delivery of therapeutic drugs or genes.

## Introduction

Hepatocellular carcinoma (HCC), is the third leading cause of cancer death in China (Chen et al., 2016). Radiofrequency ablation (RFA) which considered to be a valid local treatment method with curative intent and shows a comparable overall outcome to that of liver resection when patients with HCCs smaller than 3 cm in diameter (N’Kontchou et al., 2009; Kang et al., 2015). However, one major cause of insufficient RFA (IRFA) is the uncertain ablation margin, which may lead to local recurrence with a more aggressive phenotype and worse prognosis (Kim et al., 2010; Wang et al., 2013; Liu et al., 2015; Shady et al., 2016; Sotirchos et al., 2016; Dai et al., 2017; Zhang et al., 2019).

Some researchers found that colony-stimulating factor 1 receptor (CSF-1R) expression and tumor associated macrophage (TAM) density (CSF-1 receptor, CSF-1R or CD68) in the adjacent liver tissues are associated with patient survival after resection of HCC (Zhu et al., 2008; Jia et al., 2010; Kong et al., 2013). CSF-1R is highly expressed by monocytes (precursors of macrophages) and TAMs which support tumor cell proliferation, motility, and drug resistance (Lewis and Pollard, 2006; Pyonteck et al., 2013). CSF-1R and macrophages are the front line of defense to prevent tumor growth. The peritumoral liver tissue, which possessed of abundant CSF-1R, plays an opposite role in anti-tumor effect by providing a fertile environment for metastasis (Qian and Pollard, 2010). A high density of CSF-1R in peritumoral liver tissue, but not in tumor tissue, was associated with poor survival and a high incidence of metastasis after resection of the primary tumor (Zhu et al., 2008; Nandi et al., 2013). Leftin et al., 2019 confirmed that macrophage-targeted inhibition of CSF-1R by immunotherapy inhibits macrophage accumulation and slows mammary tumor growth *in vivo*. Thus, CSF-1R might be a feasible target for molecular imaging of HCC.

Ultrasound molecular imaging can provide high specificity and sensitivity imaging as it combines the advantages of ultrasound contrast agents (UCAs). UCAs can targeted with ligands such as antibodies or other proteins to detect expression of cancer-specific molecular markers (Jiang et al., 2016; Li et al., 2018; Wang et al., 2018). Unfortunately, traditional UCAs composed of microbubbles with a diameter about several micrometers, which cannot penetrate through the vasculature and have the short circulation time, which has constrained the advancement of ultrasound molecular imaging (Krupka et al., 2010; Wang et al., 2010). Nanobubbles (NBs, <1000 nm) were then introduced as a contrast agent enhancer in ultrasound imaging. However, NBs may decrease the echogenicity under clinical ultrasound (Sheeran et al., 2013). So it extremely challenging to fabricate not only small, highly echogenic particles but also can provide new, paradigm shifting applications of ultrasound agents in diagnosis and therapy (theranostics; Guvener et al., 2017; Tang et al., 2017; Liu et al., 2019).

**SCHEME 1:**
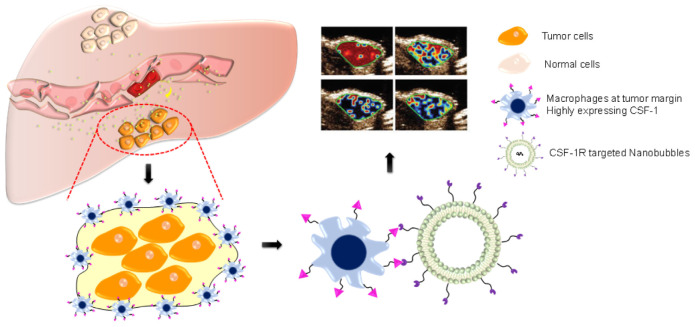
After radiofrequency ablation (RFA), macrophages are highly condensed at the tumor margin, highly expressing CSF-1R. By using nanobubbles targeting CSF-1R (NB_CSF1R_), we developed an ultrasound-guided CSF-1R specific targeted real-time monitoring of tumor margin. NB_CSF1R_ could penetrate through the vascular and adhered to the macrophages, which can provide ultrasound molecular imaging to reveal the accurate margin of HCC.

Herein, to address the above shortcomings, we designed a novel CSF-1R targeted nanobubble (NB_CSF–1R_) and characterized its properties *in vitro* and *in vivo*. We also investigated the specificity and efficacy of the nanobubbles (NB_CTRL_ and NB_CSF–1R_) against HCC xenograft tumors and IRFA models to evaluate the feasibility of using NB_CSF–1R_ in the clinical diagnosis of HCC margin (Scheme 1).

## Materials and Methods

### Materials

1,2-distearoyl-sn-glycero-3-phosphocholine (DSPC) and 1,2-dipalmitoyl-sn-glycero- 3-phosphoethanolamine (DPPE) were purchased from Avanti Polar Lipids, Inc. (Alabaster, AL, United States). Polyethylene glycol (PEG4000) was purchased from Aladdin Limited Company (Shanghai, China). 1,2-distearoyl-sn-glycero-3-phosphoethanolamine-N-[biotinyl(polyethylene glycol)-2000] (DSPE-PEG_2000_-Biotin) was purchased from A.V.T. Pharmaceutical Co., Ltd. (Shanghai, China). 4′,6-diamidino-2-phenylindole (DAPI) and 1,1′-dioctadecyl-3,3,3′,3′-tetramethylindocarbocyanineperchlorate (DiI) were obtained from Beyotime (Haimen, China). Octafluoropropane (C_3_F_8_) was purchased from Guangzhou Walter (Guangzhou, China). Antibodies for flow cytometric analysis or Western blot of CSF-1R were purchased from Novus International, Inc. (St Charles, MO, United States). Antibodies for immunohistochemistry (IHC) or immunofluorescence (IF) for CSF-1R were obtained from Abcam (Cambridge, MA, United States). Other chemicals and reagents were of analytical grade. Phorbol 12-myristate 13-acetate (PMA) was purchased from Sigma-Aldrich (St. Louis, MO, United States).

### Cells

Human monocyte THP-1 was purchased from Sun Yat-sen University Cell Bank. SMMC-7721 and HepG2 human liver cancer cell lines were kindly donated by the Radiology Department, Sun Yat-sen Memorial Hospital. The H22 cell line was obtained from Procell Life Science & Technology Co., Ltd. (Wuhan, China). Hepa1-6 mouse liver cancer cell lines were purchased from Guangzhou Genebio Biotechnology Co., Ltd. (Guangzhou, China). SMMC-7721, HepG2, and Hepa1-6 cells were cultured in Dulbecco’s modified Eagle’s medium (DMEM, GIBCO Gaithersburg, MD, United States) and supplemented with high glucose and 10% fetal bovine serum (FBS, GIBCO) at 37°C with 5% CO_2_. THP-1 and H22 were cultured separately in RPMI 1640 (GIBCO) and supplemented with 10% FBS at 37°C with 5% CO2. Macrophages were obtained from induction of THP-1 cells by 100 ng/ml of PMA for 24 h.

### Animals

All animal procedures were performed in accordance with the Guidelines for Care and Use of Laboratory Animals of Sun Yat-sen University. Experiments were reviewed and approved (NO. SYSU-IACUC-2018-000179) by the Ethics Committee of Sun Yat-sen Memorial Hospital and Ethics Committee of Zhongshan School of Medicine (ZSSOM) on Laboratory Animal Care, Sun Yat-sen University (Guangdong, China).

### Patients and Tissue Samples

Primary hepatocellular carcinomas were obtained from 30 patients at Sun Yat-sen Memorial Hospital. All samples were collected with informed consent and with the approval of the Internal Review and Ethics Boards of the indicated hospitals.

### Expression of CSF-1R *in vitro*

Quantitative real-time polymerase chain reaction (qRT-PCR), fluorescence-activated cell sorting (FACS), and Western blot were used to analyze the CSF-1R presentation in different cells. The following primers were used: human CSF-1R: forward (5′- > 3′) AGCGATAGGTCCCCGTGTTTT, reverse (5′- > 3′) CAACGGTGACCTTGCGATGTG, murine CSF-1R: forward (5′- > 3′) CAGGGTCCAAGGTCCAGTAGG, reverse (5′- > 3′) TGGTTGTAGAGCCGGGTGAAA. Macrophages and SMMC-7721 cells were seeded into six-well plates at 5 × 10^5^ cells/well in 2 mL of medium for 12 h. Cells were collected and each sample was divided into two tubes. One tube was incubated with anti-CSF-1R antibodies for 30 min and then rinsed with phosphate-buffered saline (PBS) one time. Then, the samples were incubated with PE-conjugated anti-mouse IgG for 20 min and rinsed with PBS. The fluorescence intensity in the macrophages and SMMC-7721 cells was calculated by Flow Cytometry (Beckman Coulter, Fullerton, CA, United States).

### Expression of CSF-1 *in vivo*

Immunohistochemistry (IHC) analysis of human liver cancer tissue and peritumor tissue adjacent to tumor (about 10 mm) was performed. Procedures for IHC analysis of CSF-1R (anti-CSF-1R antibody, 1:200 dilution, Novus International, Inc., United States) were performed. Procedures for IHC analysis of CSF-1R (anti-CSF-1R antibody, ab215441, 1:100 dilution, Abcam, Cambridge, MA, United States) were performed in two random fields in tumor tissue and peritumor tissue for each slide. The quantification of stained cells was analyzed by Image-Pro Plus. The slides were observed by using a light microscope (ECLIPSE 80i, Nikon, Japan).

### Preparation of the Nanobubbles

Nanobubbles were prepared according to our previous studies (Jiang et al., 2016; Zhou et al., 2019). Briefly, a homogenous mixture containing DSPE-PEG_2000_-biotin, DSPE-PEG_2000_, DSPC, and DPPE at a mole ratio of 2.5:2.5:30:10 was mixed in 15 mL chloroform. The mixture was stirred for 1 h, then vacuum dried for 2 h at 60°C using a rotary evaporator (EYELA, Tokyo, Japan). The resulting film was rehydrated with PBS and agitated for 2 h. The size of the resulting liposomes was reduced by sonication, and then C_3_F_8_ gas was injected to replace the air over the fluid to generate NBs.

The bubbles were purified by centrifugation and collected according to our previous research. Then, NBs were resuspended in PBS and stored at 4°C. For the development of fluorescent NBs, DiI-encapsulated NBs were prepared through the same method, with the addition of DiI in the initial mixture of phospholipids in chloroform. DiI-encapsulated NB_CSF–1R_ were observed by inverted fluorescence microscope (Olympus IX73, Japan) and Western Blot. Excitation wavelength of Dil is 549 nm and the emission wavelength is 565 nm.

### Western Blot Analysis

In order to determine the success of CSF-1R onto NBs surface, SDS-PAGE and Western blot were used. An 8% SDS–polyacrylamide gel was loaded with NB_CTRL_, NB_CSF–1R_, and CSF-1R_mAb_ (Novus International, Inc., United States) and electrophoresed under reducing condition for 2 h at 60 mV and for an additional 180 min at 300 mA. The gel was then transferred to a membrane and blocked using 5% skim milk. After blocking, the membranes were Horseradish peroxidase (HRP)-conjugated goat anti-rabbit IgG (1:2000 dilution; Santa Cruz Biotechnology, Santa Cruz, CA, United States) was used as the secondary antibody. Protein signals were detected using a chemiluminescence system (New Life Science Products, Boston, MA, United States).

### Preparation of NB_CSF–1R_

*In vitro* CSF-1R_mAbs_ was biotinylated using the EZLink NHS-Biotin Kit (Muralidhara et al., 2019; Wang et al., 2019). Biotinylated CSF-1R_mAb_ was bound to the NBs (NB_CTRL_) by linking the biotin groups of CSF-1R_mAb_ and DSPE-PEG_2000_-biotin on the NBs with Streptavidin. Briefly, nanobubbles was mixed with biotinylated CSF-1R_mAb_ using a DSPE-PEG_2000_-biotin:CSF-1R_mAb_: Streptavidin molar ratio of 30:1:15, then incubated at 4°C for 8 h (NB_CSF–1R_). To remove the excess free CSF-1R_mAb_, the upper layer of the suspension was centrifuged three times (1000 rpm, 5 min) and stored at 4°C. To determine the success of conjugation, the CSF-1R_mAb_ was labeled with fluorescein isothiocyanate (FITC) and co-localization of the CSF-1R_mAb_ with CSF-1R were confirmed by fluorescence microscope.

### Characterization of NB_CTRL_ and NB_CSF–1R_

#### Size, Zeta, Concentration, TEM, and Stability Test

The mean diameter and Zeta potential of NB_CTRL_, and NB_CSF–1R_ were measured using a Malvern Zetasizer Nano (Malvern Instruments, Ltd., United Kingdom). Their morphology was detected by scanning electron microscopy (SEM, SU8020, Hitachi, Japan). The concentration of NBs was measured with a Coulter counter (Multisizer 4e, United States) according to Liu et al. (2019).

The long-term stability test of NB_CSF–1R_ were confirmed by using a Vevo 2100 small animal imaging device with a frequency of 20 MHz, in a static state. NB_CSF–1R_ was diluted from 100 to 10,000 times. The contrast imaging was then observed for each sample. To determine the long-term stability of NB_CSF–1R_, the above experiments were repeated in samples that had been stored for 1, 3, or 6 months at 4°C. As a control, Sonovue^®^ was suspended at the same concentration.

### Cytotoxicity Analysis

Macrophages were induced from THP-1 cells. Approximately 5 × 10^6^ cells were cultured with 100 ng/ml PMA for 24 h at 37°C with 5% CO_2_. SMMC-7721 cells and macrophages were separately inoculated into 96-well plates at 3000 cells/well for 12 h. The same volume of fresh media with various concentrations NB_CSF–1R_ were incubated with the cells for an additional 24 h, the concentration of NB_CSF–1R_ ranging from 2 × 10^3^ to 2 × 10^8^ bubbles/ml. Then, 10 μL CCK-8 reagent in 100 μL fresh medium replaced, and incubated for an additional 2 h. The plates were gently shook for 5 min, and Infinite F200 multimode plate reader (Spectra Max M5, Molecular Devices) was used to test the absorbance of each well at 450 nm. All experiments were conducted in triplicate.

### *In vitro* Targeting Ability of NB_CSF–1R_

SMMC-7721 and macrophages were seeded into confocal dishes at 1 × 10^5^ cells/dish and grown for 24 h at 37°C with 5% CO_2_. The cells were then rinsed gently with PBS three times at room temperature, 4% paraformaldehyde was added for 5 min, then cells were gently rinsed again with PBS three times. Then, 1 ml of PBS containing 0.5% Triton X-100 was added for 5 min and rinsed with PBS three times. The remaining steps were performed in the dark: added 100 μL of diluted phalloidin solution (5 μL of phalloidin solution to 200 μL of PBS containing 0.1% BSA) to cover the cells in the center of the confocal dish; incubated for 30 min; added 200 μL DiI labeled NB_CSF–1R_ or NB_CTRL_ to the center of the confocal dish and incubated for 2 h at 37°C with 5% CO_2_; added 200 μl of 100 ng/ml DAPI solution and incubated for 5 min; gently rinsed 5 times with PBS to remove the unbound CSF-1R. The cells were observed under a laser confocal microscope to observe the fluorescence distribution of the cytoskeleton and the NB_CSF–1R_, and the specific targeting of the NB_CSF–1R_ to the CSF-1 was observed.

### *In vivo* Contrast-Enhanced Imaging

To generate tumors, approximately 1 × 10^7^ SMMC-7721 cells in 100 μL of single-cell suspension was injected into 5–6-week-old male BALB/c nude mice (*n* = 30, five animals/group) in the right hind legs, subcutaneously (*s.c.*). The mean maximum tumor size at ultrasound was about 10 mm. In this experiment, the mice were divided into six groups (*n* = 30). Group 1 = NB_CTRL_, Group 2 = NB_CSF–1R_, Group 3 = Sonovue, Group 4 = NB_CTRL_ + IRFA, Group 5 = NB_CSF–1R_ + IRFA, Group 6 = Sonovue^®^ + IRFA. During imaging, Mice were kept warm using a heated stage and a heat lamp, and anesthesia at 2% isoflurane in oxygen at 2 L/min during imaging. Mechanically, the contrast enhanced imaging can only generated while enveloped bubbles undergo compression and expansion. In this experiment, negative blank (PBS) was not included as PBS was unable to generate ultrasound intensity. Three groups received radiofrequency ablation to simulate IRFA models, which was performed using a bipolar RFA device (Radionics, INC, Burlington, MA, United States), radiofrequency energy about 30 watts for 30 s. One group of the xenograft tumor models and one group of the residual cancer models received NB_CSF–1R_. Mice were anesthetized with isoflurane by full anesthesia machine and placed on a warm pad. Approximately, 4 × 10^7^ NB_CSF–1R_ was injected through caudal veins. The ultrasound contrast parameters were: (Visual Sonics, Vevo 2100) Transducer: MS-250; Frequency: 20 MHz; Imaging Mode: Non-linear Contrast Mode; Dynamic Range: 30 dB; Overall Contrast Gain: 45 dB; Output Power: 4%. NB_CTRL_ and Sonovue^®^ were injected through caudal veins similarly. VevoCQ software was used to export the image of ultrasound molecular imaging (USMI) signal, and then observe the differential targeted enhancement distribution in the region of interest (green contour).

### Statistical Analysis

For data analysis, Statistical Package for the Social Sciences (SPSS) version-21 (SPSS, Inc., Chicago, IL, United States) was used. GraphPad Prism version 5.00 (GraphPad Software, Inc., San Diego, CA, United States) was used to generate figures. *p* < 0.05 was considered statistically significant. Data from the experiments was expressed as mean ± SD for technical replicates and the mean ± SEM for biological replicates. ANOVA was performed to compare differences between multiple groups and Differences in continuous variables were analyzed by Student’s *t*-test to compare two groups. A non-parametric test of two paired samples was analyzed by Wilcoxon Signed Rank Test.

## Results

### CSF-1R Expression *in vitro* and *in vivo*

**FIGURE 1:**
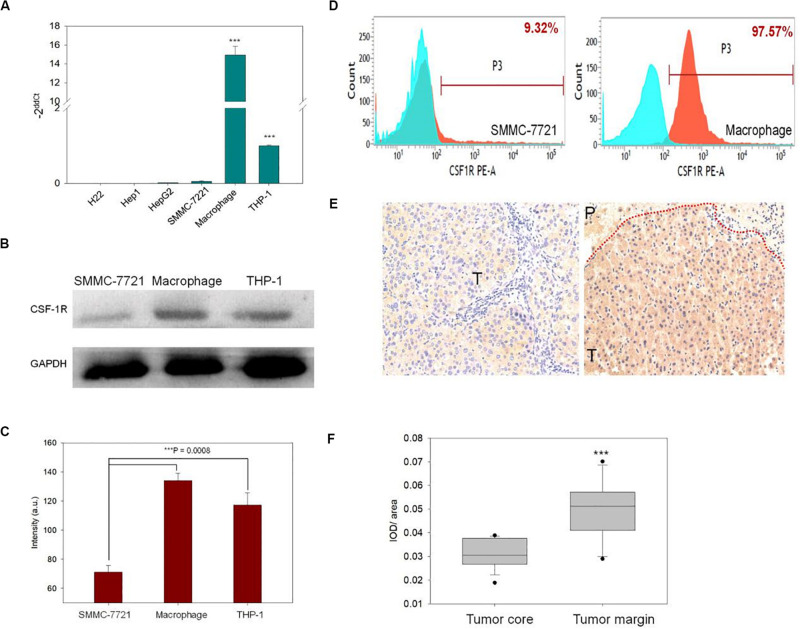
**(A)** qRT-PCR was used to determine the expression of colony-stimulating factor 1 receptor (CSF-1R) in different cell lines. **(B)** Western blot was used to access the protein level of CSF-1R in macrophages, THP-1, and SMMC-7721. **(C)** Showed the CSF-1R expression intensity in macrophages, THP-1, and SMMC-7721, macrophages showed the highest intensity than THP-1 and SMMC-7721, (***indicates *p* < 0.01). **(D)** The expression level of CSF-1R in macrophages and SMMC-7721 were detected by fluorescence-activated cell sorting (FACS) blue indicated the control group and, red indiacted the experiment group. **(E)** IHC verified the expression distribution of CSF-1R in human liver cancer tissues (the red line indicated the boundary of hepatocellular carcinoma (HCC). (P, peritumoral, T, tumor). **(F)** Semi-quantitatively analyzed the expression of CSF-1R by image-pro plus (IPP) software (***indicates *p* < 0.01).

To verify the expression of CSF-1R *in vitro*, qRT-PCR, Flow Cytometry, and Western blot were carried out. As seen in Figure 1A, qRT-PCR analysis revealed that the expression of CSF-1R mRNA is significantly higher in macrophages as compared to H22, SMMC-7721, HepG2, Hepa1-6, and THP-1 (*p* = 0.05). We then proceed to select a mouse originated cell line SMMC-7721 for consideration of *in vivo* experiments. Western blot analysis also confirmed that the protein level of CSF-1R is overexpressed in macrophages and THP-1, while minimally expressed in SMMC-7721 cells (Figure 1B). Comparison of CSF-1R intensity showed a significantly greater extent of expression within macrophages (macrophage: intensity of 143.75 ± 4.2 a.u.; THP-1: 103.02 ± 3.4 a.u.; SMMC-7721: 78.36 ± 3.4 a.u.; *p* < 0.001; Figure 1C). Quantification analysis using FACS indicated that 97.57% of macrophages are CSF-1R positive compared to 9.32% of SMMC-7721 cells (Figure 1D).

Immunohistochemistry analysis was carried out to confirm the expression of CSF-1R in HCC patients. As seen in Figure 1E, CSF-1R deposits were detected in the peritumoral tissues of carcinoma *in situ* in human HCC (Figure 1E). The counts of positive CSF-1R differed significantly between the normal tissue and the margin (*p* < 0.05; Figure 1F).

### NPs Synthesis and Characterization Particle Surface Modification

**FIGURE 2:**
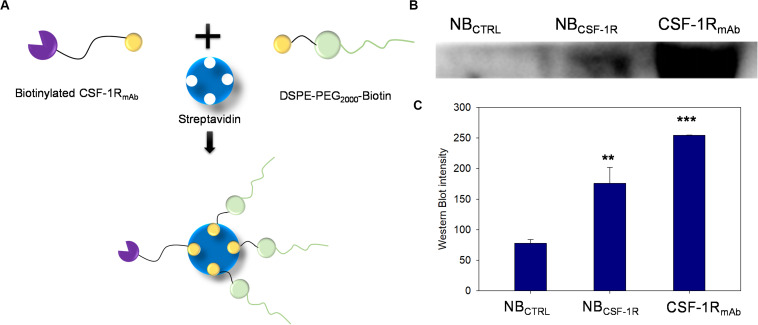
**(A)** The process of synthesis of nanobubble CSF-1R (NB_CSF–1R_) through the streptavidin/biotin chemical effect, CSF-1R_mAb_ was biotinylated and then conjunction to the 1,2-distearoyl-sn-glycero-3-phosphoethanolamine-N-[biotinyl(polyethylene glycol)-2000] (DSPE-PEG_2000_)-Biotin. **(B)** Western blot showed that the NB_CSF–1R_ band was at approximately the same position as CSF-1R_mAb_, and the NB_CTRL_ showed no expression of CSF-1R. **(C)** Showed the Western blot intensity of NB_CSF–1R,_ CSF-1R _mAb,_ NB_CTRL_ (**indicates *p* < 0.05, ***indicates *p* < 0.001).

Figure 2A shows the schematic illustration of NB_CSF–1R_ fabrication through Streptavidin/biotin interaction. Western blot showed that the band intensity of CSF-1R attached on NB_CSF–1R_ was similar with CSF-1R input, while NB_CTRL_ showed no indication of CSF-1R band (Figure 2B), indicating that CSF-1R successfully conjugated with the NB_CSF–1R_ specifically (*p* < 0.005; Figure 2C).

**FIGURE 3:**
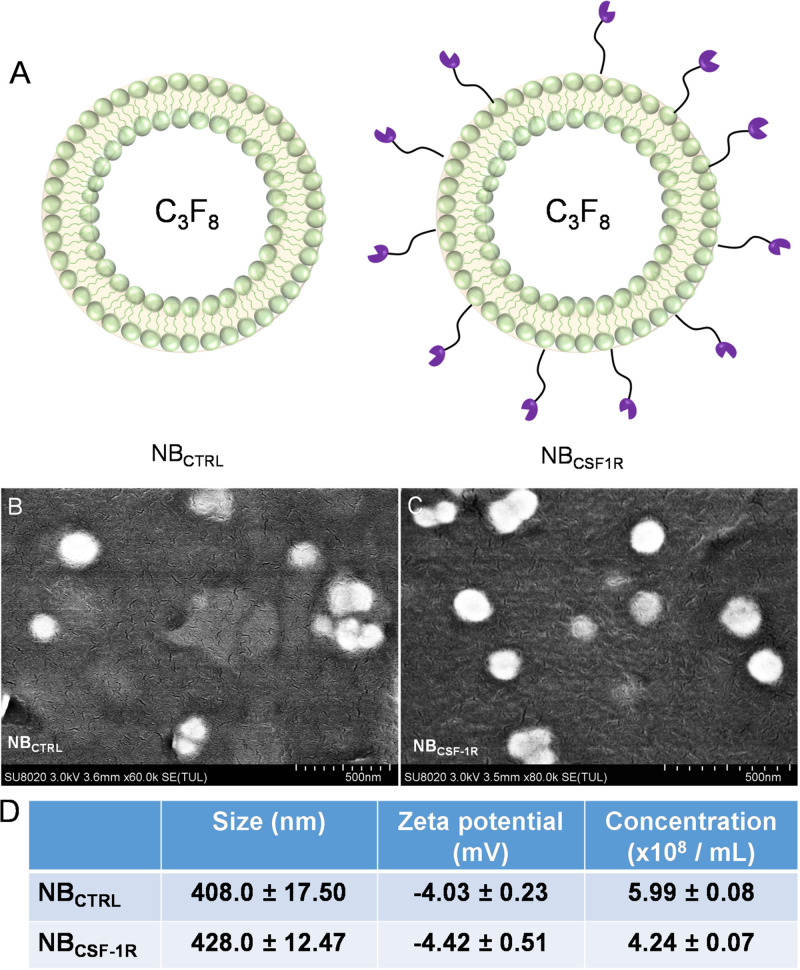
**(A)** Showed the structure of the NB_CTRL_ and NB_CSF–1R_, **(B)** scanning electron microscopy (SEM) image of NB_CTRL_ with a scale bar of 500 nm **(C)** SEM image of NB_CSF–1R_ with a scale bar of 500 nm. **(D)** Summary of the average size, and zeta potential of NB_CTRL_ and NB_CSF–1R_ as measured by dynamic laser scattering. Concentration of NBs were measured by Coulter counter. Data represent mean ± SD (*n* = 5).

Figure 3A depicted the two NBs synthesized, the non-targeted NB_CTRL_ and the targeted NB_CSF–1R_. The morphologies of NB_CTRL_ and NB_CSF–1R_ were observed by SEM. As shown in Figures 3B,C, NB_CTRL_ and NB_CSF–1R_ were spherical, uniformed in size and had distinct shell structures. The physical properties of NB_CTRL_ and NB_CSF–1R_ are summarized in Figure 3D. Dynamic laser scattering (DLS) analysis indicated that the average hydrodynamic size of NB_CTRL_ and NB_CSF–1R_ was (408.0 ± 17.5) nm and (428.0 ± 12.47) nm, respectively. Zeta potential values showed that NB_CTRL_ was with charge of −4.03 ± 0.23 mV, and NB_CSF–1R_ was −4.42 ± 0.51 mV. The concentrations of NB_CTRL_ and NB_CSF–1R_ were (5.99 ± 0.08) × 10^8^ bubbles/mL (*n* = 5) and (4.24 ± 0.07) × 10^8^ bubbles/mL (*n* = 5), respectively.

### CSF-1R-Binding Efficiency to the NBs

**FIGURE 4:**
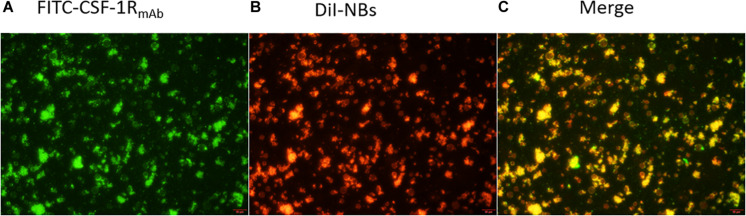
Fluorescence microscopy image of target NBs. **(A)** CSF-1R_mAb_ (FITC showed green fluorescence), **(B)** DiI-dyed (nanobubbles showed red fluorescence), and **(C)** their co-localization (merge) under fluorescence microscope, with a sale bar of 200 μm.

To illustrate the *in vitro* binding efficacy and co-localization of NB_CSF–1R_ with CSF-1R, we synthesized DiI-labeled NB_CTRL_ while CSF-1R_mAb_ were labeled with FITC. After co-incubation, the cells were observed under microscope. The green light of the FITC-labeled antibody (Figure 4A) and the red light of the DiI-labeled nanobubbles (Figure 4B) merged perfectly (Figure 4C), indicating that CSF-1R_mAb_ were successfully attached to the NBs, and could specifically target CSF-1R.

### *In vitro* Cytotoxicity and Stability of NB_CTRL_ and NB_CSF–1R_

**FIGURE 5:**
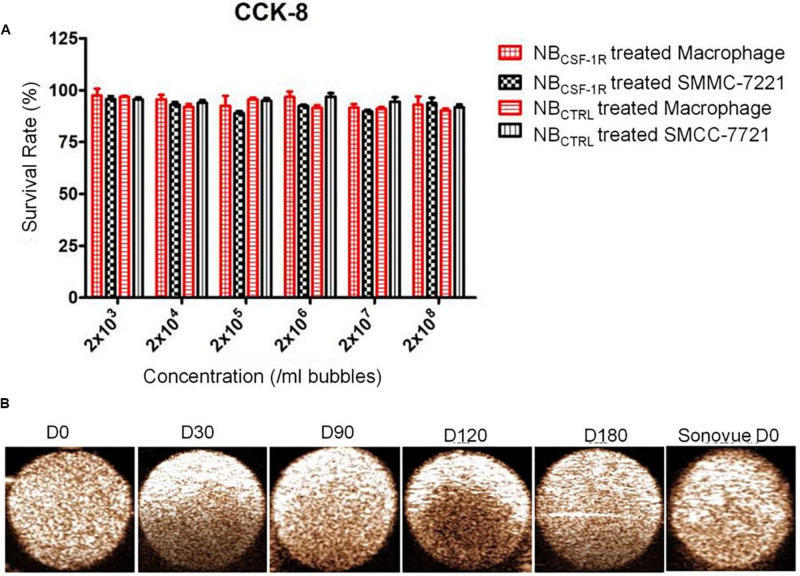
**(A)** Cell viability test for NB_CSF–1R_ determined through CCK-8. *In vitro* cytotoxicity assays using macrophage and (high CSF-1 expression) and SMMC-7721 cells (low CSF-1 expression) incubated with NB_CSF–1R_ for 24 h; there was no significant difference in the viability of macrophages and SMMC-7721. **(B)** *In vitro* ultrasound images of NB_CSF–1R_ stored for 0, 30, 90, 120, 180 days, and the Sonovue^®^ stored for 0 days as a control. Ultrasound frequency, 20 MHz.

After the induction of THP-1 cells into macrophages by 100 ng/ml PMA, the cells changed from suspension state to adherent state, and some of the cells became spindle-like, which confirmed that successful induction of THP-1 cells into macrophages. The cytotoxicity of NB_CTRL_ and NB_CSF–1R_ was evaluated using SMMC-7721 and macrophages incubated with NB_CTRL_ at five concentrations between 10^8^ and 10^3^/mL for 24 h (Figure 5A). Both SMMC-7721 and macrophages incubated with NB_CTRL_ did not show significant changes in cell viability in all concentration after 24 h of incubation. The cell viability of both SMMC-7721 cells and macrophages remained (85% after incubation with either type of NB_CTRL_, indicating they were minimally cytotoxic. These results show that NB_CTRL_ and NB_CSF–1R_ have good biocompatibility and cause minimal harm to the tested cells.

The echogenic properties of NB_CSF–1R_ were investigated in agarose gel phantom in comparison to Sonovue^®^ *in vitro*, using a Vevo 2100 small animal imaging device with a frequency of 20 MHz. The signal enhancements of NB_CSF–1R_ stored at 4°C for different periods of time (0, 30, 90, 120, and 180 days) were investigated. As indicated in Figure 5B, echogram result of NBs at Day-180 indicated no significant difference between NBs and Sonovue^®^ at Day-0 indicating that the NB_CSF–1R_ was stable.

**FIGURE 6:**
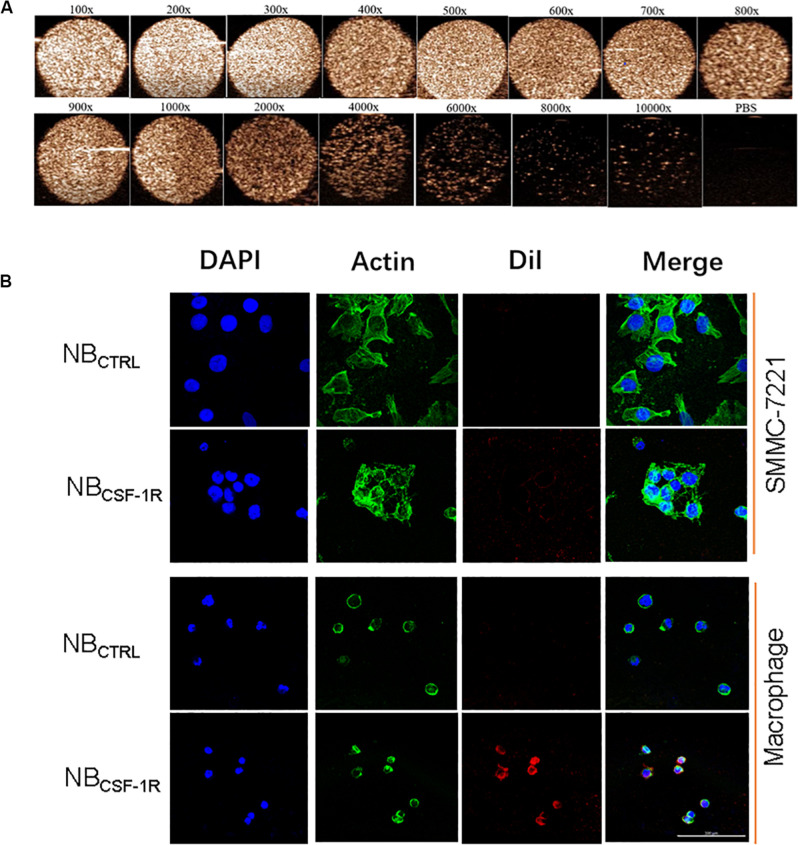
**(A)** *In vitro* ultrasound images of various concentrations of NB_CSF–1R_ dilute for different times, imaging was still viable even when diluted 2000 times. Ultrasound frequency, 20 MHz. **(B)** *In vitro*, the same quantity of NB_CSF–1R_ and NB_CTRL_ were added to SMMC-7721 and macrophages and then observed using confocal laser scanning microscopy (CLSM). There were few NB_CTRL_ adhered to SMMC-7721 and macrophages, and also few NB_CTRL_ bounded to macrophages. There were a lot NB_CSF–1R_ bounded to macrophages, and showed the specificity of targeting. With a scale bar of 100 μm.

The capability of NB_CSF–1R_ was also assessed *in vitro* using a Vevo 2100 small animal imaging device with a frequency of 20 MHz at various concentrations. Different concentrations of NB_CSF–1R_ nanoparticles (approximately 1 × 10^4^ ∼6.0 × 10^6^/bubbles of same volume, 100 μL) were evaluated in this experiment. The signal intensity decreased with the decreasing concentrations of NB_CSF–1R_ (Figure 6A). However, even when the NB_CSF–1R_ were diluted 2000 times, the signal intensity remained relatively strong.

To determine the binding ability of NB_CTRL_ and NB_CSF–1R_ in SMMC-7721 cells and macrophages, we carried out confocal laser scanning microscopy (CLSM) assay. The cytoskeletons with FITC phalloidin were green and the NBs labeled with DiI were red. As seen in Figure 6B, the red fluorescence intensity of macrophages treated with NB_CSF–1R_ was much higher than SMMC-7721 cells treated with NB_CSF–1R_, NB_CTRL_ and macrophages treated with NB_CTRL_, while minimal attachment of NB_CSF–1R_ and NB_CTRL_ were seen in SMMC-7721. This result indicates that more NB_CSF–1R_ adhered to macrophages, and demonstrated its excellent targeting ability.

### Stability and Ultrasound Sensitivity of the Targeted NBs *in vivo*

**FIGURE 7:**
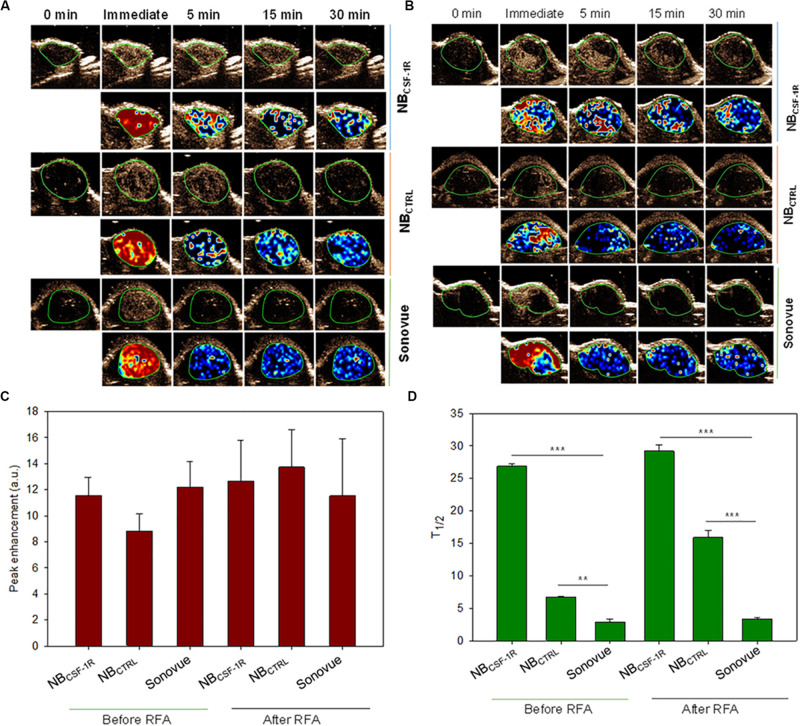
**(A)** *In vivo* tumor imaging, images were taken at the indicated time points (0, immediate, 5, 15, and 30 min) after NB_CSF–1R_, NB_CTRL_, and Sonovue^®^ were injected into tail vein of the transplanted tumors. **(B)** *In vivo* tumor imaging, images were taken at the indicated time points (0, immediate, 5, 15, and 30 min) after NB_CSF–1R_, NB_CTRL_, and Sonovue^®^ were injected into tail vein of insufficient radiofrequency ablation (IRFA) models. Perfusion imaging was also taken to show the nanobubble diffusion. **(C)** The peak intensity of NB_CS–F1R_, NB_CTRL_, and Sonovue^®^ was showed and there was no significant difference in each group. **(D)** The washout half-time in six group was show, and the NB_CSF–1R_ can provide longer imaging time in the xenograft tumor and IRFA models (**indicates *p* < 0.05, ***indicates *p* < 0.001).

*In vivo*, NB_CSF–1R_, NB_CTRL_, and Sonovue^®^ were tested in xenograft tumors and IRFA models which had been inoculated with SMMC-7721 cells (*n* = 30, 5 mice for each group). After examination, none of the mice exhibited apparent signs of distress in each group, under the same ultrasound conditions. Contrast-enhanced images of the tumors continuously exposed to ultrasound were taken at minutes 0, 5, 15, and 30 (Figures 7A,B). The peak intensity and washout half-time were compared between NB_CSF–1R_, NB_CTRL_, and Sonovue^®^ in these models (Figures 7C,D). The peak intensity of NB_CS–F1R_, NB_CTRL_, and Sonovue^®^ (Figure 7C) was 11.55 ± 1.397 a.u, 8.826 ± 1.348 a.u, 12.20 ± 1.974 a.u in the xenograft tumors, and 12.67 ± 3.126 a.u, 13.74 ± 2.878 a.u, 11.53 ± 4.401 a.u in the IRFA models (Figure 7C). There was no significant difference between the groups (Figures 7A,B). The washout half-time of NB_CSF–1R_, NB_CTRL_, and Sonovue^®^ in the xenograft tumors was 29.17 ± 1.08 min, 15.87 ± 1.05 min, 3.35 ± 0.16 min (Figure 7D), and 26.84 ± 0.44 min, 6.71 ± 0.07 min, 2.89 ± 0.44 min in IRFA models (Figure 7D). Therefore, in the xenograft tumors and IRFA models, the washout half-time (Figure 7D, *p* = 0.05) was significantly different between NB_CSF–1R_, NB_CTRL_, and Sonovue^®^. As shown in Figures 7A,B, even after 30 min, the NB_CSF–1R_ contrast agent can still enhanced efficiently in xenograft tumors and IRFA models, which implied that it has a longer circulation time *in vivo*.

**FIGURE 8:**
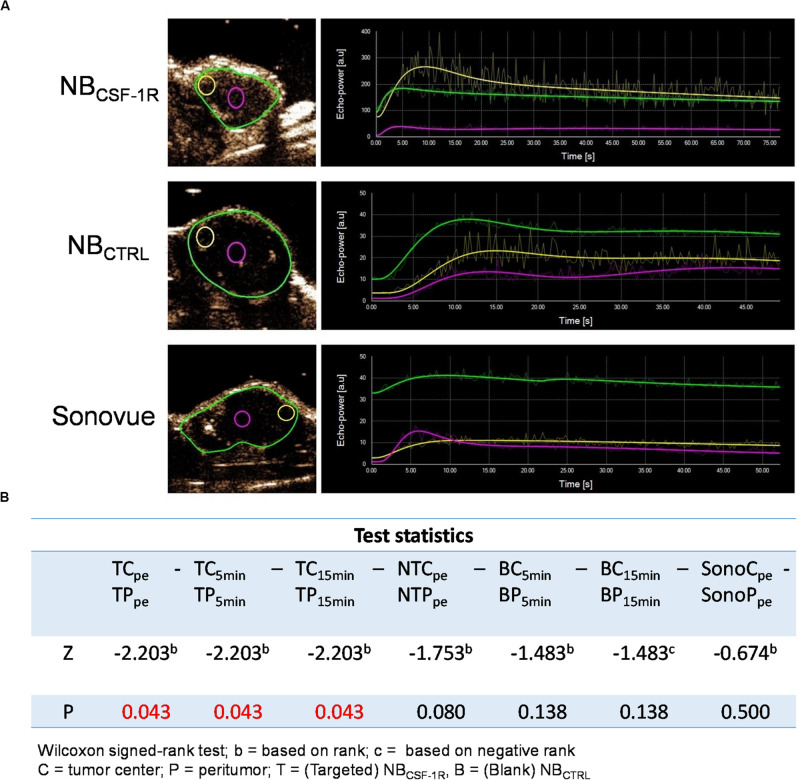
**(A)** The echo intensity fitting curve of the same area around or in the center of transplant tumor with NB_CSF–1R_, NB_CTRL_, and Sonovue^®^. The intensity of the peritumoral echo signal of the NB_CSF–1R_ was significantly higher than that of the central tissue at the peak time, 5 and 15 min (*p* = 0.05). Data was shown in panel **(B)**.

In the xenograft tumors, the echo signal intensity of NB_CSF–1R_, NB_CTRL_, and Sonovue^®^ in the peritumoral tissues and tumor center are shown (Figure 8). The results indicated that the intensity of the peritumoral echo signal of NB_CSF–1R_ was significantly higher than that of the central tissue (Figures 8A,B, *p* = 0.05) at the peak time, 5, and 15 min.

### Immunofluorescence Analysis of the Deposition of CSF-1

**FIGURE 9:**
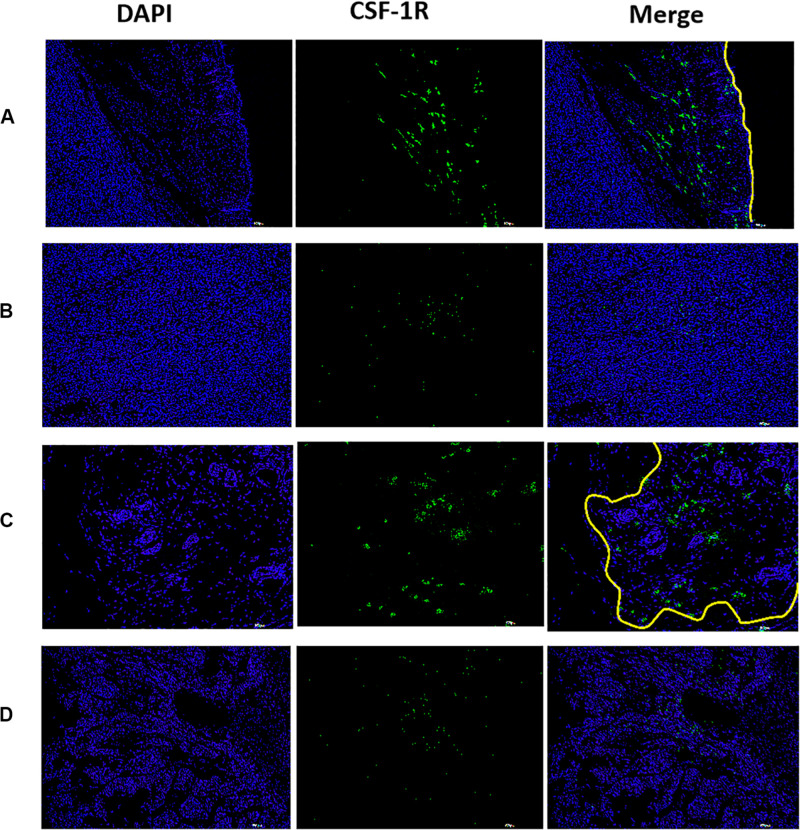
The distribution of CSF-1R in xenograft tumor observed by fluorescence immunoassay, higher fluorescence intensity was observed at the peritumor, lower fluorescence intensity was observed at the tumor center. [**(A)** boundary of the tumor, **(B)** central of tumor, **(C)** boundary of the residual tumor after IRFA, and **(D)** residual tumor after IRFA. With a scale bar of 200 μm. The yellow line showed the tumor margin].

Colony-stimulating factor 1 receptor deposits were detected at the boundary of the tumor (Figure 9A), and were also detected at boundaries of the residual tumor after IRFA (Figure 9C). However, there were few deposits detected at the center of the tumor tissue (Figure 9B) or the residual tumor tissue (Figure 9D). The fluorescence intensity at the peritumor was higher than the tumor center. Therefore, similar to the human HCC spatial infiltration profiles, CSF-1R expressed in murine HCC were also abundant at the outer margins of the tumors. These results support the potential of using CSF-1R as a cancer imaging biomarker of macrophages.

## Discussion

Researches have shown that RFA can lead to acute serologic elevation of active cytokines such as IL-6, nMDSC, and mMDSC, and a sustained high infiltration level of macrophages in the residual tumor (Shi et al., 2019; Sugimoto et al., 2019). In this study, CSF-1R was found highly expressed at the tumor boundary in patients with HCC, and also highly expressed in macrophages, but not tumor cells; making CSF-1R a feasible target. Frozen sections of the tumors revealed that macrophages were mostly located at the boundary of the xenograft tumors and residual tissue after performing IRFA.

Nanobubble CSF-1R had an average size of about 428 nm and were ultrasound-visible even at 20 MHz both *in vitro* and *in vivo*; imaging was still viable even when diluted 2000 times. Notably, NBs were administered at a low concentration compared with our previous research and other studies (Wischhusen et al., 2018), a technique which can be employed to reduce the level of background signal and modulate facilitate the comparison of heterogeneous tumor models (Wang et al., 2010, 2016).

To gain the insight functions of NB_CSF–1R_, we explored the specificity and efficiency of targeting of NBs in SMMC-7721 cells and macrophages. The results confirmed that the CSF-1R antibody could bound onto NBs efficiently; and the resulting NB_CSF–1R_ were stable and target specific. In an *in vitro* cell binding experiment, these NB_CSF–1R_ were identified to aggregate selectively surrounding macrophages but not SMMC-7721 cells, implying that the attachment of NBs to CSF-1R-positive macrophages contributes to interactions between antigen and antibody. Moreover, unconjugated NBs did not bind to macrophages, suggesting that the CSF-1R antibodies conjugating on the surface of the NBs were able to specifically recognize and improve adhesion to macrophages with high CSF-1R expression. *In vivo*, non-invasive imaging modality can be applied in extra-vascular region once NBs penetrate deep into the tumor neovasculature with a feature of a maximum pore size of approximately 380—780; this is because a basement membrane and smooth muscle absent and the intercellular space expands in cancer vasculature (Maeda, 2015).

Reduction in the size of the MBs not only decreases its echogenicity under clinical ultrasound but also cause instability (Sheeran et al., 2013). In our *in vivo* imaging experiments, however, showed that the peak intensity of NB_CSF–1R_, NB_CTRL_, and Sonovue^®^ had no statistical difference in the xenograft tumor models and IRFA models (Figure 7C). This is probably due to the fact that lipid contrast agents can produce preferable harmonic signal intensity (Postema and Schmitz, 2007), and nanoparticles could be accumulate within tumor tissue through the enhanced permeability and retention (EPR) effect and then were transformed into micro-sized echogenic bubbles (Min et al., 2016). These microbubbles at targeted tumor tissues could serve as new echogenic particles for cancer-targeting ultrasound imaging.

With the application of acoustic radiation forces (ARF) to upregulate contrast agent binding (Zhao et al., 2004), molecular ultrasound imaging is constantly improving. Frinking et al. (2012) manifested enhanced adhesion of targeted MB *in vivo* upon ARF performed in experimental models of cancer. In comparison with normal vessels, they found an increased binding of VEGFR2-targeted MB (BR55) in the vasculature of experiment (Frinking et al., 2012). In this study, in the xenograft tumor model and IRFA model, the washout half-time ratio of NB_CSF–1R_ to NBs was two times higher, and about nine times higher compared to Sonovue^®^. Furthermore, being a nanoparticle, NB_CTRL_ and NB_CSF–1R_ could accumulate at the targeted tumor tissue via the EPR effect, and NB_CSF–1R_ can abound onto higher CSF-1 expression cells effectively. The adherent NB_CSF–1R_ maintained visible for a long time, contributing to a longer persistence of enhanced contrast compared to NB_CTRL_ and Sonovue^®^. This result further verifies that the duration of contrast enhancement may be applied as an indicator for the investigation of targeted NBs enhanced imaging. The molecular imaging would also be helpful in finding the residual tumor after IRFA. With long-term stability, NB_CSF–1R_ could be used to evaluate the boundary of the tumor when performing RFA.

## Conclusion

In this study, a uniform nano-sized lipid NBs was prepared, and could successfully combined the NBs with biotinylated anti-CSF-1R. The NB_CSF–1R_ which was small and stable as well as high specificity for the molecule that is overexpressed in macrophages. We demonstrated the high specificity of our NB_CSF–1R_ on targeting CSF-1R overexpressing macrophages and HCC tumor margin. *In vitro* and *in vivo* studies demonstrated that NB_CSF–1R_ exhibited effective ultrasound imaging capabilities in evaluating the RFA response, which can be used to detect the residual HCC after RFA, opening a possibility of clinical translation of a non-invasive diagnosis method for IRFA.

## Data Availability Statement

All datasets generated for this study are included in the article/supplementary material.

## Ethics Statement

The animal study was reviewed and approved by Ethics Committee of Sun Yat-sen Memorial Hospital and Ethics Committee of Zhongshan School of Medicine (ZSSOM) on Laboratory Animal Care, Sun Yat-sen University.

## Author Contributions

HL and BZ performed animal imaging analysis. YK performed statistical analysis. PS and BL designed and oversaw all the experiments and wrote the manuscript. All authors contributed to the article and approved the submitted version.

## Conflict of Interest

The authors declare that the research was conducted in the absence of any commercial or financial relationships that could be construed as a potential conflict of interest.
